# Association of rs4516035 Polymorphism with Osteoporosis in the Southeastern Iranian Population: A Case-Control Study

**DOI:** 10.34172/jrhs.2024.138

**Published:** 2024-03-18

**Authors:** Mohammad Mehdi Yaghoobi, Azadeh Samare Gholami

**Affiliations:** ^1^Research Department of Biotechnology, Institute of Science and High Technology and Environmental Sciences, Graduate University of Advanced Technology, Kerman, Iran

**Keywords:** Bone density, Single-nucleotide polymorphism, Vitamin D3 Receptor, Pharmacogenetics

## Abstract

**Background:** Genetic polymorphisms are known to play a crucial role in the development of osteoporosis. Vitamin D_3_ regulates bone homeostasis through the vitamin D receptor (VDR). Reduced VDR activity increases osteoporosis risk.

**Study Design:** A case-control study.

**Methods:** This case-control study investigated the potential association between six single-nucleotide polymorphisms (SNPs) within the *VDR* gene (rs11568820, rs4516035, rs2228570, rs1544410, rs7975232, and rs731236) and the occurrence of osteoporosis in Kerman province. The genotypes of the SNPs were analyzed using polymerase chain reaction-restriction fragment length polymorphism, tetra primer amplification refractory mutation system-PCR, and sequencing in two groups of osteoporosis patients (n=40) and controls (n=42). Additionally, the levels of calcium and vitamin D_3_ in the serum of the patients were measured, and the in silico analysis of the VDR structure and interaction was performed using I-TASSER, ProSA, PROCHECK, GeneMANIA, GTEx, and GPS 6.0.

**Results:** None of the patients exhibited calcium or vitamin D_3_ deficiencies. Among the six SNPs, only the T allele in rs4516035, which leads to a shorter variant called VDRA, showed a significant association with susceptibility to osteoporosis (odds ratio=3.061, *P*=0.007). The in silico analysis demonstrated that the 3D structure, expression, and post-transcriptional modification of VDRA are distinct from those of the more extended variant, VDRB1. VDRB1 is upregulated in sun-exposed skin, and its interactions with its partners differ from those of VDRA.

**Conclusion:** Despite adequate vitamin D levels, the VDRA variant, which has lower activity, could increase the predisposition to osteoporosis in the studied population. These findings clarify the importance of genetic screening for personalized medicine and the effectiveness of prevention and treatment strategies.

## Background

 Osteoporosis is a widespread disease affecting the skeletal system, marked by a reduction in bone density and deterioration of bone tissue structure, leading to heightened bone fragility. The global prevalence of osteoporosis is estimated at 18.3%. The World Health Organization (WHO) estimates that there will be three million hip fractures annually by 2025. In 2020, Iran had 154,530 osteoporotic fractures, leading to 3554 deaths. It is estimated that over eight million people in Iran currently have osteoporosis.^1-4^ Factors such as gender or genetics are fixed risk factors for osteoporosis, while other factors can be modified, including dietary choices or physical inactivity. People with a genetic predisposition are more likely to develop osteoporosis, even if they have enough calcium or vitamin D_3_ in their bodies.^5,6^ Vitamin D receptor (VDR) has a significant role in the development of osteoporosis. Upon binding with vitamin D_3_, VDR forms a heterodimer with the retinoic acid receptor (RXRA), translocates into the nucleus, and regulates target gene expression and many biological functions.^7^

 Some single-nucleotide polymorphisms (SNPs) in VDR fundamentally influence its function, thus leading to osteoporosis.^8^ The presence of the T allele in rs11568820 leads to greater calcium absorption and increases bone mass.^9^ T/C alleles in rs4516035 encode VDRA and VDRB1, respectively, with different transcriptional activities.^8^ In FokI (rs2228570), the T allele increases the risk of osteoporosis. The three SNPs BsmI (rs1544410), ApaI (rs7975232), and TaqI (rs731236) are near the 3’UTR region of the VDR and usually show a linkage disequilibrium (LD).^9^

 The frequency of genetic polymorphisms and susceptibility to osteoporosis varies between ethnic groups. Najmi Varzaneh et al observed that the FF genotype at FokI was higher in the elderly than in the control group in Tehran.^10^ Pouresmaeili et al reported that the frequency of the AA genotype at BsmI was higher in osteoporotic patients. In contrast, the frequency of the GG genotype was higher in normal women in Tehran.^11^ Mohammadi et al found a significant association between FokI polymorphism and osteoporosis in postmenopausal women in Sanandaj.^12^ A recent report has also confirmed an association between the Tt genotype of the rs731236 and the EE genotype of the rs4516035 with an increased risk of osteopenia/osteoporosis in women with type 2 diabetes in northwest Iran.^13^

 The above studies have primarily focused on an SNP within a limited population. To address the limitations of prior research, the current study investigated the association between six SNPs in the *VDR* gene (rs11568820, rs4516035, rs2228570, rs1544410, rs7975232, and rs731236) and osteoporosis in the southeastern region of Iran. Certain SNPs may impact osteoporosis development. Examining different regions can help us understand how genetics influence osteoporosis in Iran. Studying these variations can lead to personalized medical approaches for the effective prevention and treatment of osteoporosis.

## Methods

###  Sampling and clinical evaluation 

 From December 2019 to July 2021, a case-control study was conducted in Kerman province, where peripheral blood samples were collected from patients and control groups. All participants were randomly selected from the population and provided written, informed consent. The participants provided information such as age, gender, drug usage, and smoking habits. The sample size was calculated using Epi Info^TM^ (CDC, USA, version 7.2.4.0), considering a confidence level of 90% and a power of 80%. Ethical approval was obtained from the National Research Ethics Committee on November 30, 2019 (approval ID: IR.UK.VETMED.REC.1398.026). The research followed the Strengthening the Reporting of Observational Studies in Epidemiology guidelines.^14^

 The bone mineral density (BMD) was measured at the lumbar spine and hip using a Norland XR-36 Densitometer Machine (Swissray International, Inc.) in the Radiology Department of the Samen Al-Hojaj Charitable Institution in Kerman. BMD results were reported as T- and Z-scores. Individuals recommended by their physicians for BMD measurement with a T-score > 1, as per WHO guidelines, were included in the study.^1^ Nonetheless, patients were excluded if their osteoporosis was due to a congenital or genetic disease, motor disability, or cancer, or if they were from the same family. Calcium and cholecalciferol (25(OH)D_3_) levels in patients’ serum were measured by enzyme-linked immunosorbent assay.^15^ The control group consisted of healthy adult men and women with no history of osteoporosis of the same ethnicity. The sole distinction between the two groups was the presence or absence of osteoporosis, while both groups were as similar as possible in terms of gender, age, and the like. Blood samples from all members of both groups were collected in ethylenediaminetetraacetic acid tubes and used for DNA extraction.

###  DNA extraction

 According to the instructions, genomic DNA was extracted from 200 µL of the peripheral blood of all members of the two groups with a Blood Genomic DNA Extraction Kit (Pars Tous Company, Mashhad, Iran). The quality and quantity of DNA were assessed by running agarose gel electrophoresis and measuring absorption at 260 nm and 280 nm.

###  Polymerase chain reaction

 Seven pairs of primers for six SNPs were designed using Primer-BLAST and synthesized by Pishgam Biotech or Sinaclon Company (Tehran, Iran). The restriction fragment length polymorphism method was utilized for loci with cleavage sites for restriction endonuclease enzymes. The tetra primer amplification refractory mutation system-polymerase chain reaction (T-ARMS-PCR) method was employed for each GATA and CDX2 loci (Table 1).

**Table 1:** The sequence of primers designed for six SNPs and the length of PCR products

**SNP/Primers**	**PCR product length (bp)**
CDX2 (rs11568820)	
F1: ACCCATAATAAGAAATAAGTTTTTAC R1: AGGATAGAGAAAATAATAGAAAACA	109
F2: CGTTAAGTTCAGAAAGATTAATTC R2: ATATTCCTGAGTAAACTAGGTCACAA	238
GATA (rs4516035)	
F1: GACCTCCTTTAGCCAGGGAAGAT R1: GTGAGGGGCTGGTTATGATGT	240
F2: GGCGTGAGGGGAAAAGATA R2: CTGTAAGAGGCGAATAGCAATG	314
FokI (rs2228570)	
F: GGGCTCCCTTCATGGAAAC R: GCTGAGCCAGCTATGTAGG	434
ApaI (rs7975232)	
F: GAATGGGCTGGGTGGATAGG	592
TaqI (rs731236)	
R: ATGCACGGAGAAGTCACTGG	592
BsmI (rs1544410)	
F: TCACCTCTAACCAGCGGAAG R: GCAACCTGAAGGGAGACGTAG	386

*Note*. SNP: Single-nucleotide polymorphism; PCR: Polymerase chain reaction.

 PCR was conducted on the DNA samples of both patients and controls for all six SNP variants. The PCR was performed in a final volume of 10 μL, including 5 μL of Taq DNA Polymerase 2X Master Mix RED kit (Amplicon, Odense, Denmark), 400 ng DNA template, and 300 nM of each primer in the Biometra TAdvanced thermocycler (Analytik Jena GmbH, Germany). The thermal program included 95 °C for five minutes and then 35 cycles of 95 °C for 25 seconds, 59‒64 °C (depending on the primer) for 20‒25 seconds, and 72 °C for 30 seconds. Then, 72 °C was applied for five minutes as the final extension step. PCR conditions were optimized to prevent nonspecific amplification.

###  Digestion with restriction endonuclease enzymes

 PCR products (0.2‒0.5 μg) were cut in a final volume of 30 μL by the restriction endonuclease enzymes FokI, ApaI, TaqI, and BsmI (Thermo Fisher Scientific, USA) for 16 hours. The digestion products were run on 2% agarose gel to determine the number and length of bands. The gels were stained with GelRed (Biotium, Belgium) and photographed with G:BOX HR (Syngene, Cambridge, UK).

###  DNA sequencing and protein sequence analysis

 To check the validity of PCR data, fourteen samples of CDX2 and GATA fragments were randomly selected from either case or control groups, amplified by outside primers, and sequenced by Pishgam Biotech Company (Tehran, Iran). The sequencing results were analyzed in the Chromas program (version 2.6.6, Technelysium, Australia).

###  In silico analysis

 The 3D structure of VDR variants was analyzed at the I-TASSER (https://zhanggroup.org), ProSA (https://prosa.services.came.sbg.ac.at/prosa.php), VERIFY 3D, ERRAT, and PROCHECK (Ramachandran plot) at https://saves.mbi.ucla.edu.^16^ A particular view of models was conducted using the PDB and PyMOL. The 3D structure of RXR/VDR was modeled by the existing crystal structures fitting between the two main parts of the complex. VDR’s physical interaction with other proteins was derived from GeneMANIA (https://genemania.org). Phosphorylation sites on VDRB1 were explored on GPS 6.0 at https://gps.biocuckoo.cn. The expression of VDR and its variants in different tissues was derived from the GTEx Portal (https://gtexportal.org/home).

###  Statistical analysis

 A combination of quantitative and qualitative approaches was used in the data analysis. The two groups were allocated considering potential confounding variables such as age, gender, and drug usage to minimize bias. Individuals were randomly assigned to groups based on the presence or absence of osteoporosis. Calcium and vitamin D3 amounts, T-score, and Z-scorewere analyzed using the IBM SPSS Statistics program (version 25, 2017). The data were presented as means ± standard deviations (SD). Comparisons between the two groups were made using an independent sample *t *test.

 The frequency of genotypes and alleles in both groups was analyzed by the Pearson chi-square test in the SPSS program. The Hardy-Weinberg equilibrium (HWE) was evaluated using the chi-square (χ^2^) test in Microsoft Excel 2019. The LD between the SNP loci was determined using the SNPStats website. Minor allele frequency (MAF) was calculated using the following formula:

*MAF = (2 × A allele)/(2 × (A allele + a allele))*

 Binary logistic regression was used to investigate the association of age, gender, each genotype, and adjusted variables with the risk of osteoporosis in the SPSS program. The odds ratio (OR) with a 95% confidence interval (CI) was computed for all variables in complete genotype, dominant, recessive, and multiplicative models.^17^ A *P* value < 0.05 was considered statisticallysignificant.

## Results

###  Clinical evaluation data

 During the 19 months of sampling, 40 patients (31 females and seven males) were included in this study. Two cases inadvertently did not report their gender. The control group included 42 normal people (38 females and 4 males, with a mean age of 41 years). All patients had osteopenia or osteoporosis based on their T- or Z-scores. None of the confounding variables, such as medication, smoking habits, and age, were statistically significant in either the case or control group. In addition, no previous bone fractures were reported in patients. Table 2 presents a significant decrease (*P*= 0.019) in the average age of female patients (52.61 years) compared to male patients (64.86 years). This finding suggests that females develop osteoporosis at a younger age than males. No statistically significant difference was observed in the mean spine T-score among male and female subjects (*P*= 0.088). Nevertheless, the results demonstrated that the femur T-score for males was statistically inferior to that of females (*P*= 0.004).

**Table 2:** The mean spine T score, femur T score, age, calcium, and vitamin D_3 _in male and female patients

**Variables**	**Mean**	**SD**	***P *** **value**
Spine T-score (g/cm^2^, young matched)			0.088
Female	-1.85	0.66	
Male	-1.37	0.59	
Femur T-score (g/cm^2^, young matched)			0.004
Female	-1.39	0.93	
Male	-2.55	0.60	
Age (years)			0.019
Female	52.61	12.66	
Male	64.86	7.47	
Calcium (mg/DL)			0.027
Female	9.23	0.38	
Male	9.60	0.32	
Vitamin D_3_ (ng/mL)			0.667
Female	35.83	20.52	
Male	39.32	10.91	

*Note*. SD: Standard deviation.

 Furthermore, the mean T-score for the femur in all males was found to be below -2.5, indicating the presence of severe osteoporosis (Table 2). Thus, our data proves that women are more susceptible to vertebral osteoporosis, whereas men are more prone to femoral osteoporosis. The normal distribution of calcium and vitamin D_3_ and spine and femur T-scores in both genders was confirmed in the SPSS program.

 Interestingly, the amount of calcium and vitamin D_3_ in all patients, regardless of gender, fell within the normal range (Table 2).^15^ No significant difference was evident between the two genders’ vitamin D_3_. Nonetheless, it is worth noting that women’s average calcium concentration was significantly lower than men’s (*P*= 0.027).

###  Genotyping

 The distribution of genotypes in control and patient groups was analyzed following PCR and digestion of the six SNPs for all samples. Fortunately, the tetra-ARMS-PCR results coincided with the outcomes of the sequencing analysis. The frequency of genotypes for all SNPs (except for CDX2) corresponded to the expected values based on the HWE (*P*> 0.05 in all SNPs, except CDX2; Table 3). Our findings indicated that among the six SNPs, there was a significant difference in the frequency of genotypes and alleles for GATA between the patient and control groups (*P*= 0.060). The most remarkable discovery was the statistically significant association between the TT genotype in GATA and the risk of osteoporosis (OR = 3.061, *P*= 0.007). No significant associations were observed between the other five SNPs and the risk of osteoporosis (Table 4). The percentage of C and T alleles in the population was 27% and 73%, respectively. The MAF for the C allele was reported to be 18‒48% in other populations.^18^.

**Table 3:** The frequency of alleles and genotypes for all six SNPs in the population, along with their HWE chi-square test results and corresponding *P* values

**SNP**	**Allele** **frequency**	**Genotype**	**Observed** **frequency**	**Expected** **frequency**	**HWE Chi**^2^	**HWE ** ***P*** ** value**
rs11568820 (CDX2)	T = 114 C = 46	TT	13	6.61	12.1542007	0.00049
		TC	20	32.78		
		CC	47	40.61		
rs4516035 (GATA)	C = 44 T = 118	CC	5	5.98	0.30004736	0.58385
		TC	34	32.05		
		TT	42	42.98		
FokI (rs2228570)	f = 37 F = 125	ff (AA)	5	4.23	0.23856624	0.62524
		Ff (AG)	27	28.55		
		FF(GG)	49	48.23		
TaqI (rs731236)	t = 55 T = 105	tt (GG)	10	9.45	0.07346189	0.78636
		Tt (AG)	35	36.09		
		TT (AA)	35	34.45		
ApaI (rs7975232)	a = 63 A = 99	aa (CC)	11	12.25	0.34154157	0.55894
		Aa (CA)	41	38.50		
		AA (AA)	29	30.25		
BsmI (rs1544410)	b = 75 B = 45	bb (CC)	22	23.44	0.62696296	0.42847
		Bb (CA)	31	28.13		
		BB (AA)	7	8.44		

*Note*. SNP: Single-nucleotide polymorphism; HWE: Hardy-Weinberg Equilibrium.

**Table 4:** The frequency of genotypes and alleles of the six SNPs, their chi-square and *P* value, OR, and *P* value calculated by binary logistic regression

**SNP**	**Genotype/** **allele**	**Frequency of genotypes/alleles**		***P*** ** value**	**OR (95% CI)**	***P*** ** value**
		**Patients**	**Controls**			
rs11568820 (CDX2)	TT	6	7	0.952	1.092 (0.610, 1.953)	0.767
	TC	10	10			
	CC	24	23			
	T	22	24	0.727		
	C	58	56			
rs4516035 (GATA)	CC	0	5	0.015	3.061 (1.366, 6.864)	0.007
	CT	1	20			
	TT	26	16			
	C	14	30	0.006		
	T	66	52			
FokI (rs2228570)	ff	4	1	0.026	1.356 (0.657, 2.799)	0.410
	Ff	8	19			
	FF	28	21			
	f	16	21	0.395		
	F	64	61			
TaqI (rs731236)	tt	4	6	0.816	1.216 (0.637, 2.322)	0.553
	Tt	17	18			
	TT	18	17			
	t	25	30	0.546		
	T	53	52			
ApaI (rs7975232)	aa	5	6	0.738	0.906 (0.471, 1.742)	0.767
	Aa	22	19			
	AA	13	16			
	a	32	31	0.774		
	A	48	51			
BsmI (rs1544410)	bb	11	11	0.041	1 (0.451, 2.217)	1.000
	Bb	23	8			
	BB	2	5			
	b	45	30	1.000		
	B	27	18			

*Note*. SNP: Single-nucleotide polymorphism; OR: Odds ratio; CI: Confidence interval.

###  Linkage disequilibrium analysis of the vitamin D receptor gene

 Genotype data from patients and control groups were used to create an LD plot. ApaI, BsmI, and TaqI were found to be in the same LD block with a *P* value of 0. CDX2 and GATA were also in the same LD block with a *P* value of 0.0012. The OR was calculated for the GATA/CDX2 block, showing a significant association with osteoporosis, with an OR of 1.316 and a *P* value of 0.018. No significant LD was found between the other SNPs. In addition to the six SNPs, the OR was also computed for gender and age. There was no correlation between gender and osteoporosis (OR = 2.032, *P*= 0.292). However, the likelihood of developing the disease increased with age (OR = 1.1, *P*< 0.001). The adjusted OR for all six SNPs, along with gender, demonstrated that the TT genotype in the GATA locus significantly increased the risk of osteoporosis (OR = 6.423, *P*= 0.004).

###  In silico analysis

**Figure 1:**
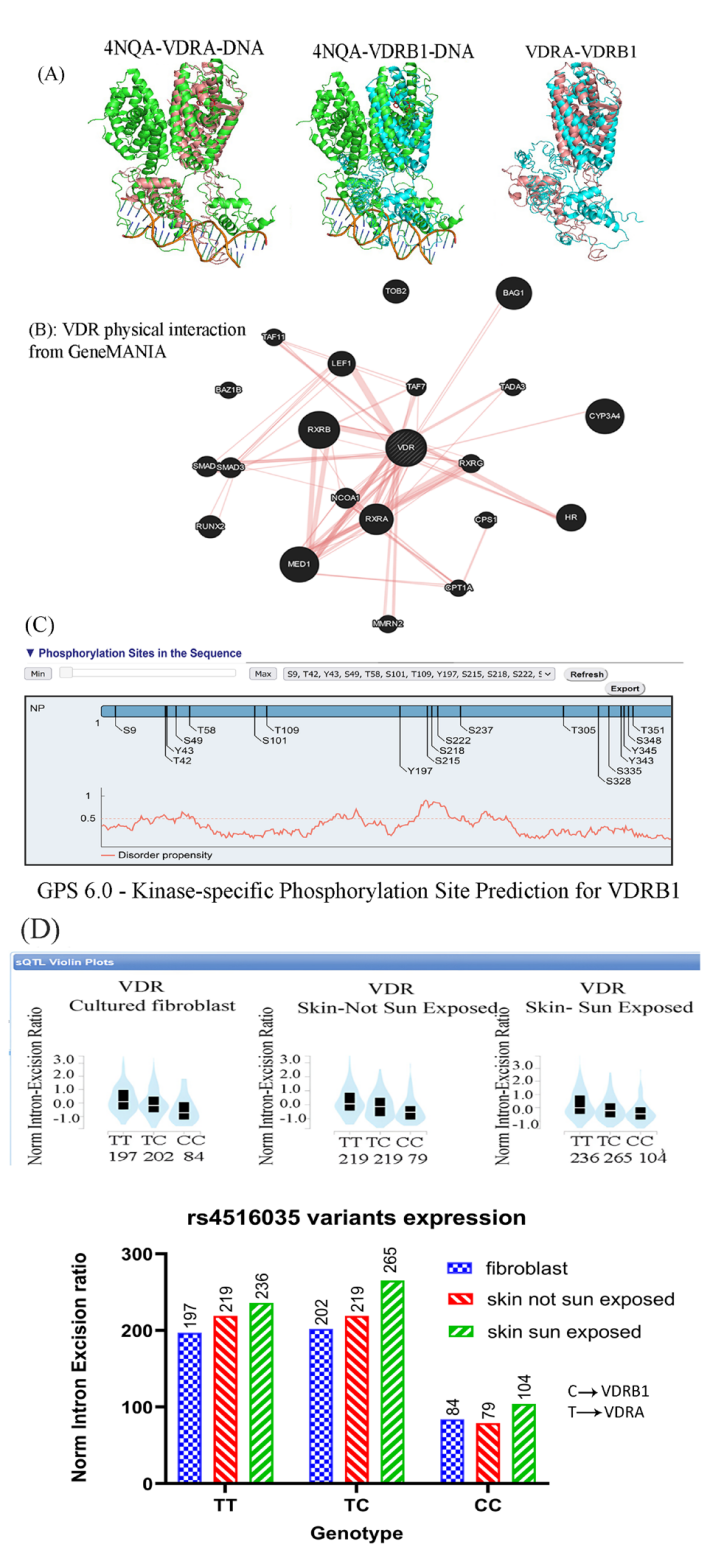
In silico analysis of the VDR protein: **(A)** I-TASSER and PyMOL analysis of VDRA (Pink) and VDRB1 (bBue) With RXR (4NQA, Green) showing an extra domain at the N-terminal of VDRB1, presumably affecting its interaction with RXA and DNA, **(B)** VRD interaction derived from GeneMANIA displaying a strong interaction with its main partner, RXR, and other proteins, such as MED1, **(C)** Kinase-specific phosphorylation predicting four new phosphorylation sites (S9, T42, Y43, and S49) on VDRB1, and **(D)** rs4516035 expression in the culture fibroblast and skin cells (Sun-exposed and not Sun-exposed). *Note*. VDR: Vitamin D receptor. The CC genotype product (VDRB1) is expressed less than VDRA in all cells. However, its expression increased in skin cells following sun exposure

 The 3D structure analysis revealed an additional domain at the N-terminal region, which affects its interaction with RXR (4NQA) and its binding to the DNA double helix (Figure 1A). The GeneMANIA output showed a strong interaction between VDR and members of the RXRs (Figure 1B). The extra domain contains specific phosphorylation-susceptible residues (S9, T42, Y43, and S49; Figure 1C).

**Figure 2:**
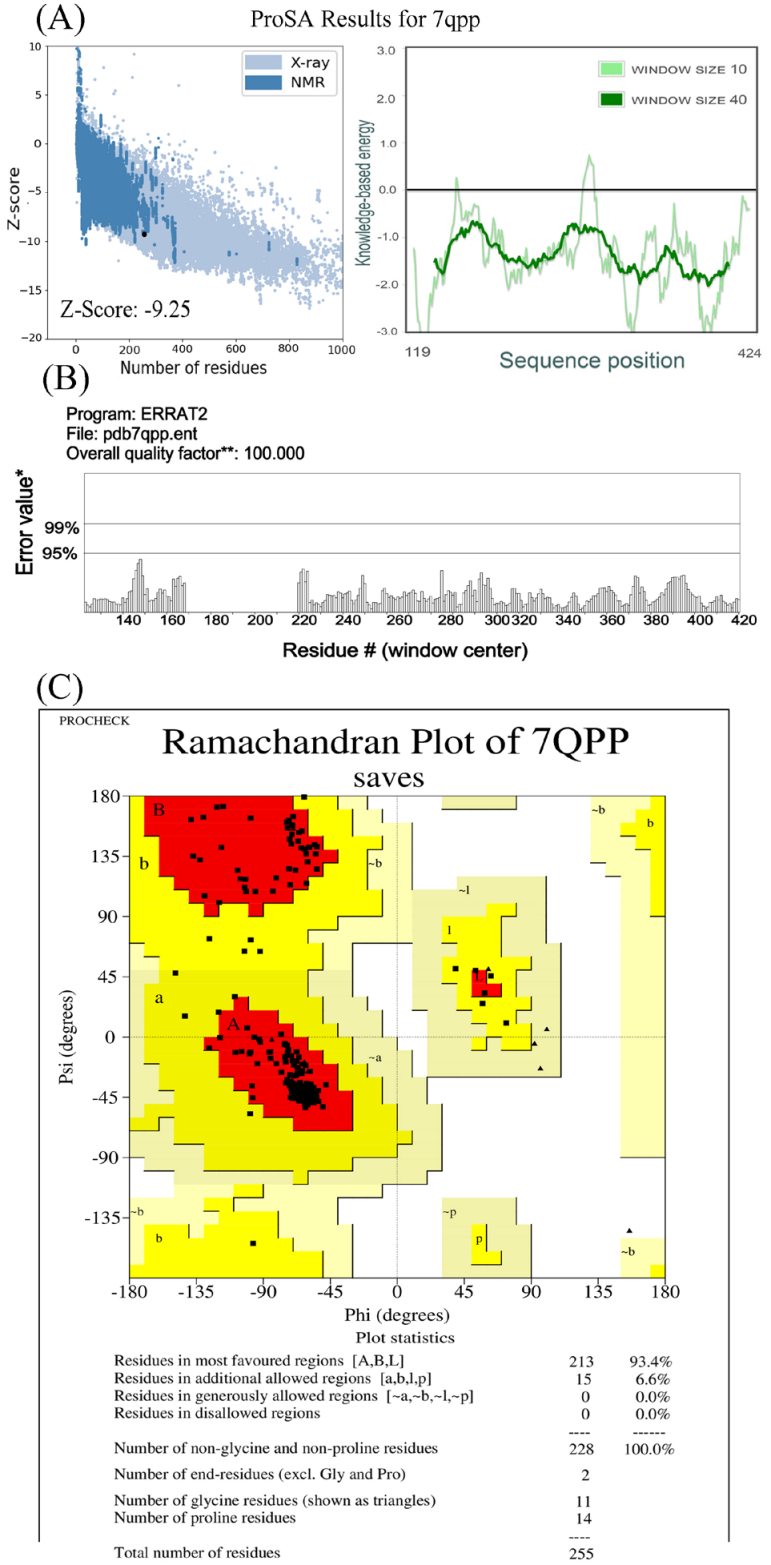
Analysis of VDR Protein Structure Using ProSA **(A)**, ERRAT **(B)**, and Ramachandran Plot **(C)**. *Note*. A negative score of -9.25 suggests that the protein falls within a natural protein score range. The non-positive residual score indicates no flaws in the input structure. ERRAT analysis gave a quality score of 100, indicating high quality. The Ramachandran plot shows 93.4% of residues in the most favorable regions, with none in disallowed regions

 The analysis of the bulk tissue expression of VDR demonstrated high expression of VDR in the skin, small intestine, and colon. Interestingly, there was a remarkable difference in the expression of the rs4516035 variants (VDRA and VDRB1) in cultured fibroblasts, skin cells not exposed to sun, and sun-exposed skin cells. The expression of VDRB1 was significantly less than that of the VDRA or TC alleles (*P*= 0.0005 and *P*= 0.0003, respectively). Based on the results, the expression of VDRB1 was statistically increased in skin cells exposed to the sun (*P*= 0.032, Figure 1D). The ProSA analysis of VDR (7QPP) represented a Z-score of -9.25, within the range for native proteins of similar size. Additionally, the plot of residue scores illustrated a negative value, indicating no problematic or erroneous parts of the input structure (Figure 2A). The ERRAT report further confirmed the high overall quality of the structure, with a quality factor of 100 (Figure 2B). Moreover, the Ramachandran plot depicted that 93.4% of the residues reside in the most favored regions, while no residues were found in the disallowed regions (Figure 2C).

## Discussion

 The present study was designed to determine the association between the six well-known SNPs in the *VDR* gene and osteoporosis. Surprisingly, none of the patients were calcium or vitamin D_3_ deficient. Likewise, none of them declared taking vitamin D or calcium supplements previously. This finding indicates that they developed osteoporosis despite normal calcium and vitamin D_3_ levels, confirming that genetic factors probably play a crucial role in the development of osteoporosis in these cases.

 The EPI program calculated 40 samples in each group. The global coronavirus disease 19 outbreak caused a drastic decrease in the number of patients referred to the clinic. Nevertheless, it was attempted to collect the minimum required samples with great effort. In epidemiology-genetic studies, HWE indicates reliable data on polymorphism studies and disease association. In this study, the presence of equilibrium for rs4516035 and four other loci shows enough sample size and valid genotyping data. However, the absence of equilibrium for the CDX2 locus may be due to random sample selection. One out of every 20 tested markers will have a *P* value of less than 0.05 by chance.^17,19^ Therefore, there is no confounding due to population stratification.

 In this study, only the GATA polymorphism (rs4516035) had a significant association with the incidence of osteoporosis (Table 4). As in case-control studies, the genotype and allele data were analyzed by full genotype, dominant, recessive, and multiplicative models.^17^ Again, only the GATA frequency demonstrated a significant association with the risk of osteoporosis. Our findings are somewhat surprising, given that an association between the other SNPs, such as TaqI, BsmI, ApaI, and FokI, was reported in other countries.^20-22^

 The T allele in rs4516035 uses a start codon at exon 1a, producing the VDRA with 427 residues. The C allele adds two exons (1c and 1d) to the mature mRNA and 50 amino acids to the N-terminal of the protein.^8,23^ Our data confirmed that the TT genotype and T allele (VDRA) significantly increase the risk of osteoporosis. The DNA binding and nuclear localization domains of VDR are at its N-terminal. Adding 50 amino acids to the N-terminal could potentially influence binding to the target sequence as well as its interaction with its partner RXR (Figure 1). The lack of crystal and 3D structure of the VDRB1 protein in databases such as Protein Data Bank does not allow more analysis of its interaction with other proteins. The current structure of VDRB1 in databases is a prediction and is not based on purified protein. Further experimental investigations can uncover the disparities in the functionality of VDRA and VDRB1. Using PROSA and the Ramachandran plot, the VDR structure analysis confirmed that this protein structure is possible without hindrances. The emergence of novel sites for post-translational modifications, such as phosphorylation, within the N-terminal region of VDRB1 can affect its functionality and interaction with its partners, such as RXR.

 The VDRB1 is expressed in the small intestine, duodenum, colon, and kidneys, while its response to vitamin D_3_ differs from that of the VDRA, indicating potential tissue and ligand specificity. VDRB1 is localized in distinct foci within the nucleus without a ligand, whereas VDRA shows a uniform distribution. This distinct localization of VDRB1 with cofactors in specific areas of the nucleus may facilitate a prompt response following ligand uptake. The transcriptional activity of the two isoforms varies depending on the type of ligand, promoter, and type of cell. In some cases, the transcriptional activity of VDRB1 exceeded that of VDRA.^23,24^ The analysis of the GTEx portal revealed that the expression of VDRB1 is lower than that of VDRA in fibroblast and skin cells. However, sunlight increases VDRB1 expression in skin cells. Surprisingly, sunlight is more effective than oral vitamin D in preventing osteoporosis.^25^ Considering the absence of calcium and vitamin D deficiency in the patients, osteoporosis may be caused by less activity of the VDRA variant in their tissues. A recent study in northwest Iran has demonstrated a strong association between rs4516035 and osteoporosis in diabetic women. While they only found this SNP in women with diabetes, we found it in all individuals with osteoporosis. This suggests that this variant may increase the risk of both diabetes and osteoporosis ^13^.

 Some polymorphisms in VDR influence the efficacy of vitamin D_3_ supplementation in preventing vertebral fractures among women in New Zealand.^26^ Similarly, Brazilian investigators have observed that administering calcium and vitamin D supplements is less efficacious in individuals with the TT genotype in GATA than those with the TC genotype. Consequently, the TT genotype confers a diminished protective effect on BMD despite an augmented intake of calcium and vitamin D.^27^ Likewise, Tomei et al have recently reported that some SNPs in vitamin D-related genes have a prominent role in response to vitamin D supplementation.^28^ These data exemplify that polymorphism in the GATA locus influences individuals’ susceptibility to osteoporosis. Further investigation is required to determine whether this polymorphism, either on its own or in conjunction with other SNPs, assumes such a role. A thorough understanding of how VDR SNPs affect the occurrence of osteoporosis and other diseases such as diabetes, cancer, and multiple sclerosis can be obtained by studying the prevalence of all SNPs throughout the nation.^29-31^

 By screening libraries of vitamin D analogs, it may be possible to identify new compounds and examine their impacts on the variants of the VDR protein in future studies.^32^ The outcomes of such investigations may ultimately lead to the discovery of analogs possessing heightened transactivation potential, thereby augmenting the functionality of the VDR in individuals with a genetic predisposition.

 A larger sample size would allow for a more specialized examination of gender differences and the distinction between young and postmenopausal females. Additionally, the limited number of samples prevented the study of SNPs’ impacts on osteoporosis in the spine or femur.

**Highlights**

- Genetic factors have significant roles in causing osteoporosis in Kerman province.
- Of the six polymorphisms in the vitamin D receptor gene, only rs4516035 is implicated in osteoporosis.
- It is recommended that genetic screening programs be implemented to manage osteoporosis.

## Conclusion

 Overall, our findings highlight the importance of the genetic polymorphism of VDR in the development of osteoporosis in the studied population. The results revealed that the observed occurrence of osteoporosis is not attributable to malnutrition but rather to the T allele at the GATA locus, which generates the less active variant, VDRA. As reported by Normando et al, giving dietary supplements to TT genotype holders may not improve their BMD.^27^

## Acknowledgments

 We would like to thank Mr. Zeinolabedini for collaborating on case identification. We also thank Mrs. Abdollahi and her colleague for collecting blood samples and measuring clinical parameters. In addition, special thanks go to Miss. Zeinalabadi for her collaboration in molecular techniques.

 Mohammad Mehdi Yaghoobi (PhD)^1*^, Azadeh Samare Gholami (MSc)^1^

## Authors’ Contribution

**Conceptualization:** Mohammad Mehdi Yaghoobi.

**Data curation:** Mohammad Mehdi Yaghoobi, Azadeh Samare Gholami.

**Formal analysis:** Mohammad Mehdi Yaghoobi, Azadeh Samare Gholami.

**Funding acquisition:** Mohammad Mehdi Yaghoobi.

**Investigation:** Mohammad Mehdi Yaghoobi, Azadeh Samare Gholami.

**Methodology:** Azadeh Samare Gholami.

**Project administration:** Mohammad Mehdi Yaghoobi.

**Resources:** Mohammad Mehdi Yaghoobi.

**Software:** Mohammad Mehdi Yaghoobi, Azadeh Samare Gholami.

**Supervision:** Mohammad Mehdi Yaghoobi.

**Validation:** Mohammad Mehdi Yaghoobi, Azadeh Samare Gholami.

**Visualization:** Azadeh Samare Gholami.

**Writing–original draft:** Mohammad Mehdi Yaghoobi, Azadeh Samare Gholami.

**Writing–review editing:** Mohammad Mehdi Yaghoobi.

## Competing Interests

 The authors have no financial or non-financial interests to disclose.

## Ethical Approval

 Ethical approval was obtained from the National Research Ethics Committee on November 30, 2019 (approval ID: IR.UK.VETMED.REC.1398.026).

## Funding

 The authors would like to acknowledge the financial support of the Institute of Science and High Technology and Environmental Sciences, Graduate University of Advanced Technology, Kerman, Iran, under grant number 98.1562.
